# The impact of lifestyle intervention on left atrial function in type 2 diabetes: results from the DIASTOLIC study

**DOI:** 10.1007/s10554-022-02578-z

**Published:** 2022-03-02

**Authors:** Aseel Alfuhied, Gaurav S. Gulsin, Lavanya Athithan, Emer M. Brady, Kelly Parke, Joseph Henson, Emma Redman, Anna-Marie Marsh, Thomas Yates, Melanie J. Davies, Gerry P. McCann, Anvesha Singh

**Affiliations:** 1 Department of Cardiovascular Sciences, University of Leicester, Leicester, UK; 2 National Institute for Health Research (NIHR) Leicester Biomedical Research Centre, Glenfield Hospital, Groby Road, Leicester, LE3 9QP UK; 3 King Saud Bin Abdulaziz University for Health Sciences, Riyadh, Kingdom of Saudi Arabia; 4 Diabetes Research Centre, NIHR Leicester Biomedical Research Centre, Leicester, UK

**Keywords:** Left atrium, Type 2 diabetes, Lifestyle, Cardiac magnetic resonance imaging

## Abstract

Aerobic exercise training and low energy diets have been shown to improve left ventricular remodelling and diastolic function in adults with type 2 diabetes (T2D), albeit with differential effects. The impact of these lifestyle interventions on left atrial (LA) function, however, has not previously been reported. The DIASTOLIC study was a prospective, randomised, open-label, blind endpoint trial, in which 90 people with obesity and T2D and no prevalent cardiovascular disease were randomised to a 12-week intervention of: (i) routine care, (ii) aerobic exercise training, or (iii) low energy (≈ 810 kcal/day) meal replacement plan (MRP). Cardiac magnetic resonance (CMR) imaging was performed pre- and post-intervention. Image analysis included LA volumes (LAV), emptying fraction (LAEF), and LA strain (LAS) corresponding to LA reservoir (LAS-r), conduit (LAS-cd), and booster pump (LAS-bp) function. 73 participants with T2D (mean age 50 ± 6 years, 62% male, body mass index (BMI) 36.1 ± 5.3 kg/m^2^) completed the trial and had analysable LA images. There was no significant change in CMR measured LA volumetric function (LAV/LAEF) in any group. The routine care group showed no significant change in BMI or LAS. In the MRP group, there were significant reductions in BMI (4.5 kg/m^2^) and a significant increase in LAS-r and LAS-bp (29.9 ± 7.0 to 32.3 ± 7.0%, p = 0.036 and 14.6 ± 5.3 to 17.2 ± 3.7%, p = 0.034). The exercise group showed a small reduction in BMI (0.49 kg/m^2^), with no significant change in LAS. Compared to routine care, weight loss via a 12-week MRP, led to improvements in LA filling and contractile function in adults with T2D and obesity. However, these within-group changes were not statistically significant on between-group comparison.

ClinicalTrials.gov Identifier: NCT02590822.

**Supplementary Information:**

The online version contains supplementary material available at 10.1007/s10554-022-02578-z.

## Introduction

Type 2 diabetes mellitus (T2D) is associated with an increased risk of heart failure (HF) [1] and is the distinct clinical entity of diabetic cardiomyopathy [2]. Diabetic cardiomyopathy is described as myocardial structural or functional abnormality, independent of underlying hypertension, coronary artery disease, or other cardiac diseases [3]. Subclinical left ventricle (LV) diastolic dysfunction is typically the earliest cardiac manifestation of diabetic cardiomyopathy that precedes the occurrence of clinically overt HF [2, 4, 5].

LV diastolic dysfunction prolongs relaxation time and diminishes LV passive filling. This leads to reliance on left atrial (LA) contraction at late diastole to achieve optimal LV stroke volume [6]. LA enlargement and dysfunction are associated with LV diastolic dysfunction severity [6–8]. T2D is also associated with LA dysfunction [9], which often precede LA dilatation [10], and is independent of LV diastolic dysfunction [11], suggesting its role in reflecting potential evidence of early diabetic cardiomyopathy.

LA strain (LAS) has recently been used to assess LA function, is less load-dependent than volumetric assessment, and could play an important role in classifying LV diastolic dysfunction [12]. LAS has been shown to be an independent predictor of cardiovascular events, superior to LA volumes (LAV) and emptying fraction (LAEF) in the general population [13], patients with HF [14] and chronic kidney disease [15]. It is also a promising non-invasive predictor of elevated LV filling pressure [16, 17]. People with T2D have impaired LA reservoir and conduit function, with increased booster-pump (LA contraction) function by both strain and volumetric assessment, compared to controls [18, 19].

Lifestyle modifications, including improved dietary intake and increased physical activity, are the first-line in and are associated with improved glycaemia, blood pressure [20, 21] and reduced the risk of cardiovascular disease [22]. These may also have a role in preventing the onset of clinical HF. Indeed, in a recently completed 12-week randomized controlled trial (DIASTOLIC study), we have shown aerobic exercise improved LV peak early diastolic strain rate (PEDSR), whereas a low-energy meal replacement plan (MRP) improved glycometabolic profiles (achieving remission of T2D in over 80%), aortic distensibility, LV concentric remodelling and body weight-corrected peak exercise capacity (VO_2_) [23].

The benefits of these lifestyle interventions on LA function in adults with T2D and obesity are not well established. Our aims for this secondary analysis were: (i) to confirm the impact of T2D and obesity on LA function, and (ii) to investigate the effect of a low-energy MRP and aerobic exercise on LAV and LAS parameters by cardiac magnetic resonance (CMR) imaging, in the DIASTOLIC cohort.

## Methodology

### Population

This is a secondary analysis of the previously published DIASTOLIC study that included T2D participants and age-, sex- and ethnicity-matched controls. This was a prospective, randomised, open-label, blind endpoint trial, to study the effects of lifestyle interventions on cardiovascular structure and function. Participants with T2D and obesity were randomly assigned to receive a 12-week intervention of: (i) routine care, (ii) aerobic exercise training, or (iii) low energy (≈ 810 kcal/day) MRP [24]. Key inclusion criteria were: age 18 to 65 years, with established T2D (duration ≥ 3 months) and body mass index (BMI) > 30 kg/m^2^ (or > 27 kg/m^2^ if South Asian). Exclusion criteria were presence of significant arrythmia (atrial fibrillation), T2D duration > 12 years, current treatment with > 3 glucose-lowering medications or insulin, history/signs/symptoms of cardiovascular disease and weight loss > 5 kg in the preceding six months. People in the control group were free of T2D, obesity, hypertension and cardiovascular disease. The DIASTOLIC study was ethically approved by the National Research Ethics Service (15/WM/0222), and all participants provided written informed consent.

Participants with T2D underwent echocardiography and CMR at baseline (prior to randomisation) and 12 weeks. The controls underwent the same investigations at baseline only. The trial protocol and main outcome data have been previously published [23, 24].

### CMR

CMR images were acquired using 1.5T MRI scanner (Siemens Aera, Erlangen, Germany), an 18-channel cardiac coil and retrospective electrocardiographic (ECG) gating, using a standardised protocol, as previously published [24]. This included long (2- and 4-chamber) and short-axis cine images using a steady-state free precession end-expiratory breath-hold sequence (typical parameters: voxel size 1.90 × 1.52 × 8 mm, temporal resolution 48 ms, TR 2.76 and TE 1.15) and late gadolinium enhancement (LGE) images at the same slice positions following administration of a total of 0.15 mmol/kg of gadolinium-based contrast agent (Gadoterate meglumine, Dotarem, Guerbet LLC, France).

### CMR image analysis

Image analysis was performed offline blinded to all participants’ details, treatment group and visit type. LV assessment was conducted by G.S.G using cmr42 version 5 (Circle Cardiovascular Imaging, Calgary, Alberta, Canada) as previously described [24], whilst all LA assessment was conducted independently by A.A using Medis v3.1 (Medical imaging systems, Leiden, the Netherlands). Image quality was graded as: 0 = not analysable, 1 = fair (artefact present but images still analysable), 2 = good (artefact present but not in the region of interest), 3 = excellent.

### Left atrial volumetric assessment

Phasic LA volumes were quantified using the biplane area length method from 2- to 4-chamber cine images [25]. After contouring LA endocardial borders excluding the pulmonary veins and the LA appendage, the LA volume curve throughout the cardiac cycle is automatically generated. The LA volume curve was used to extract LA maximal volume (LAVmax), LA minimal volume (LAVmin) and LA volume pre-atrial contraction (LAVpre-A). The maximum and minimum volumes were indexed to body surface area. LA total, passive and emptying fractions were calculated using absolute values of LA volumes as follow:

$${\text{LA total EF }}\left( {reservoir \, function} \right) \, = \,\left[ {\left( {{\text{LAVmax}} - {\text{LAVmin}}} \right)/{\text{LAVmax}}} \right]\, \times \,{1}00\% ,$$

$${\text{LA passive EF }}\left( {conduit \, function} \right) \, = \, \left[ {\left( {{\text{LAVmax}}{-}{\text{ LAVpre}} \_ {\text{A}}} \right)/{\text{LAVmax}}} \right] \, \times \,{1}00\% ,$$

$${\text{LA active EF }}\left( {booster \, pump \, function} \right) \, = \, \left[ {\left( {{\text{LAVpre}} \_ {\text{A}}{-}{\text{ LAVmin}}} \right)/{\text{LAVpre}} \_ {\text{A}}} \right] \, \times \,{1}00\% .$$

### Left atrial strain assessment

**Fig. 1:**
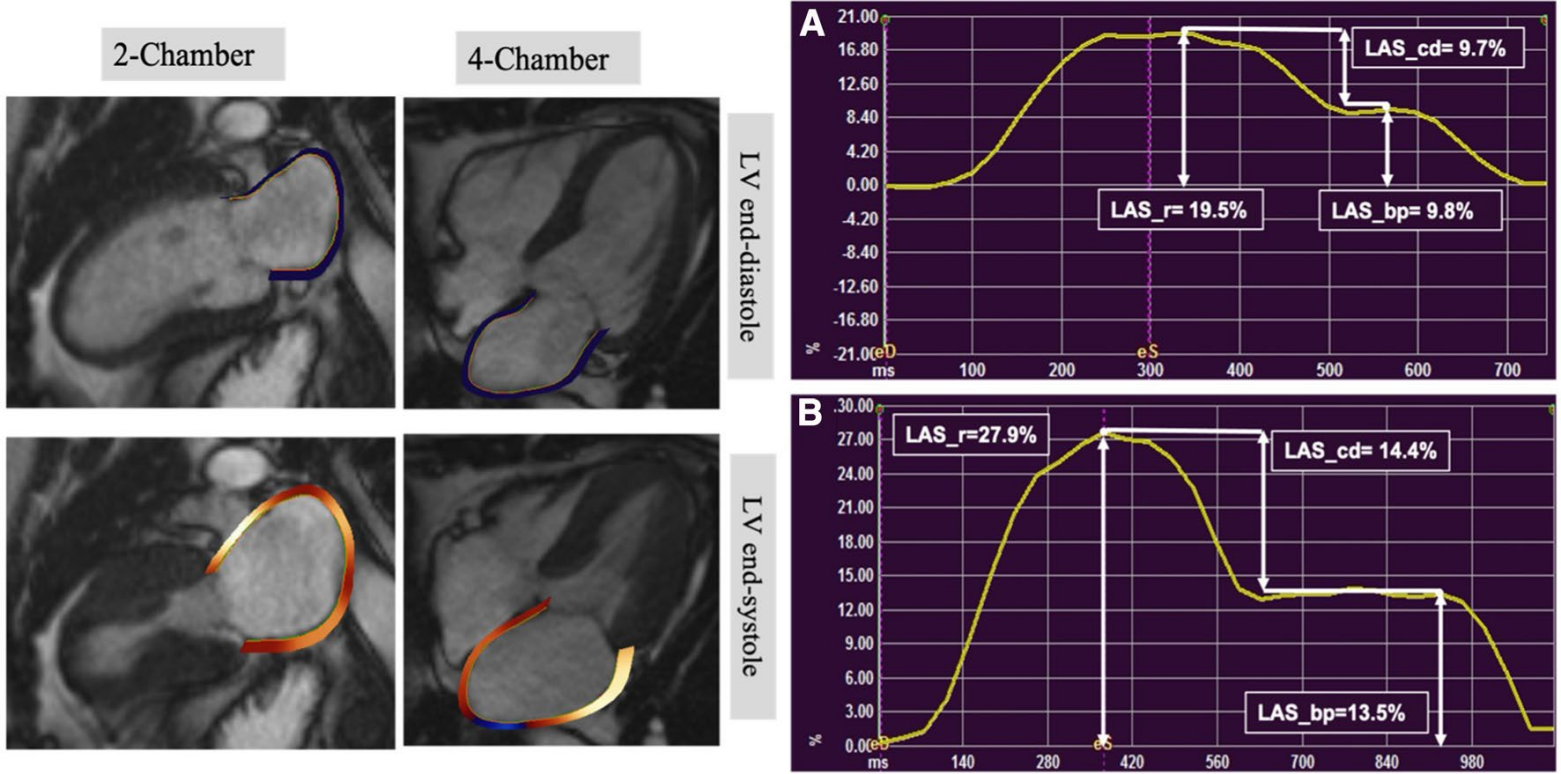
Left atrial endocardial tracking for strain assessment by feature tracking CMR, with an example of baseline and week-12 left atrial strain for a participant in MRP group. Cine 2- and 4-chamber images illustrating contoured left atrial borders at left ventricular end-diastole (upper) and left ventricular end-systole (lower). On the right, **A** LA strain curve at baseline and **B** LA strain curve at follow-up. Both curves are for the same patient and produced from tracking LA endocardium across the cardiac cycle using 4-chamber cine. LAS_cd was calculated as: LAS_r − LAS_bp (*LAS_r* LA strain at reservoir, *LAS_cd* LA strain at conduit, *LAS_bp* LA strain at booster-pump phase)

LA endocardial borders were traced at ventricular end-diastole and end-systole, excluding the LA appendage and pulmonary veins. The software automatically propagates contours to the rest of the cardiac cycle and formed LA strain curves. Manual correction of contours was performed where required. Using strain curve, LAS at *reservoir* (LAS_r) and *booster-pump* (LAS_bp) were extracted, whilst *conduit* strain (LAS_cd) was calculated as: LAS_cd = LAS_r − LAS_bp [26]. Global LAS was calculated by averaging segmental strain values from the 2- and 4-chamber cine images (Fig. 1).

### Statistical analysis

Statistical tests were performed using SPSS version 26.0 software (Statistical Package for the Social Sciences, Chicago, IL). Normality was assessed using the Shapiro–Wilk test and histograms. Numerical data are expressed as mean ± standard deviation (SD). Categorical data are expressed as counts and percentages. At baseline, differences between T2D and controls were evaluated with unpaired t-tests (continuous variables) or Chi-Square test (categorical variables). For continuous variables, One-way analysis of variance (ANOVA) was used to determine significant differences across the three trial groups at baseline.

Data were analysed using generalized linear models to compare the change from baseline to week-12 in the intervention groups relative to the routine clinical care group, adjusted for baseline value (between-group difference). The differences between baseline and week-12 values in each group were also assessed using paired t-test or Wilcoxon test as appropriate (within-group difference). The latter is included based on the novel nature of the outcomes and in order to support hypothesis generation. It should be interpreted with caution and viewed as secondary to the between-group findings. All statistical tests were two-sided, with p-value < 0.05 was considered statistically significant. Pearson’s correlation was used to assess correlation between LA function parameters. Adjustment was not made for multiple comparisons; therefore, data were viewed with caution and in relation to the overall pattern of results.

## Results

**Fig. 2:**
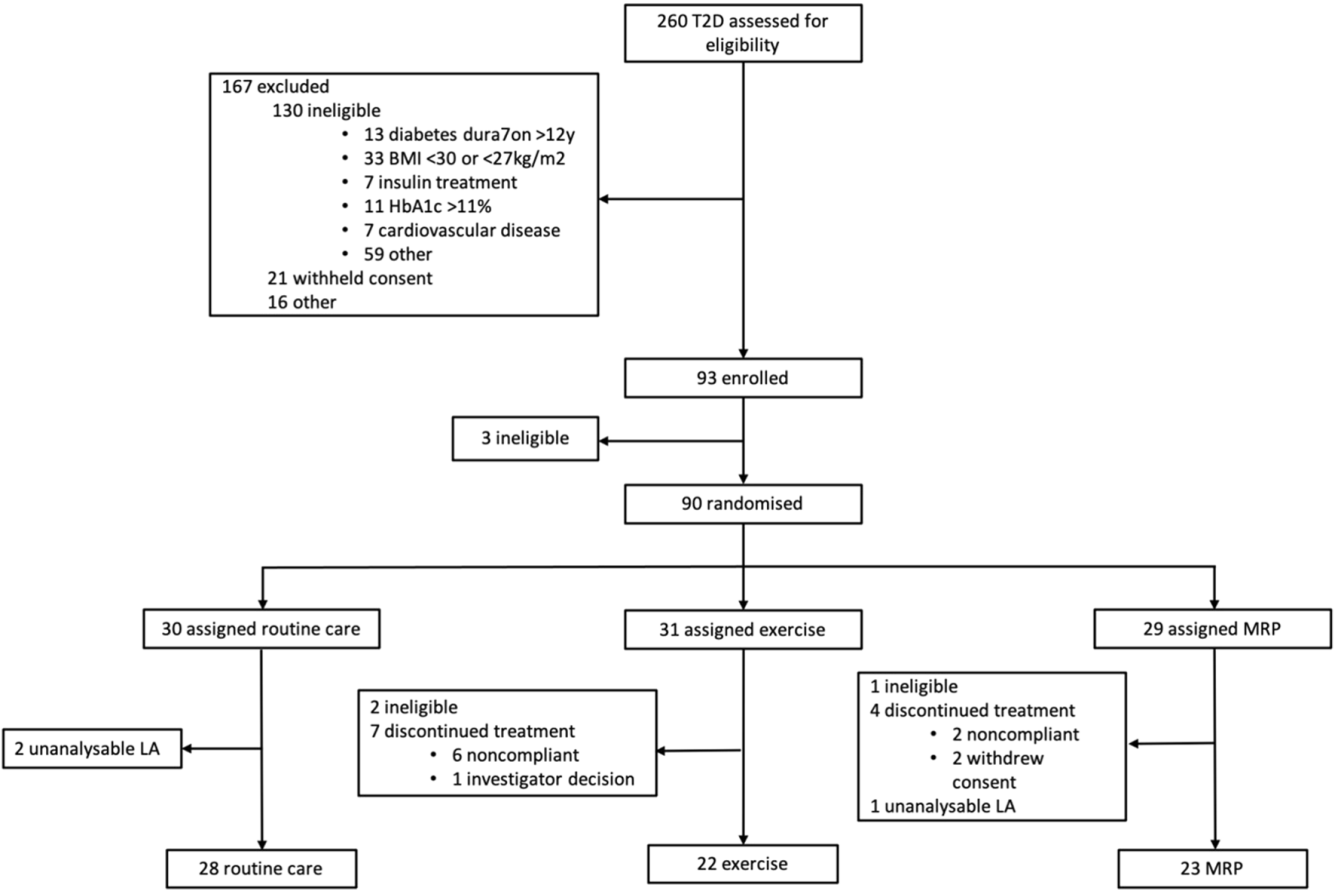
DIASTOLIC trial profile and number of participants included in the secondary analysis (*BMI* body mass index, *LA* left atrium, *MRP* meal replacement plan)

Seventy-six participants with T2D completed the trial, and 36 controls were recruited. LA analysis was not possible in 4 scans (3 T2Ds and 1 control) due to prospective ECG gating (n = 2) and LA foreshortening (n = 2). A total of 73 T2D participants (routine care (n = 28), exercise (n = 22) and MRP (n = 23)) and 35 controls were included in the analyses (Fig. 2). All CMR images were analysable (n = 181), and image quality was rated as: excellent (n = 148, 82%); good (n = 32, 17%) or fair (n = 1, 1%). At baseline, echocardiographic images for 71 (97.3%) T2D participants had analysable trans-mitral inflow velocities and 67 (91.8%) had analysable E/e’, predominantly due to body habitus.

### Baseline characteristics

Baseline demographics and clinical characteristics of participants and controls are shown in Table 1. The mean age of participants with T2D was 50 ± 6 years and 62% were men. Healthy controls were well matched for age, sex and ethnicity. Participants with T2D had higher body weight, BMI, blood pressure, heart rate and glycated haemoglobin than controls. Prevalence of hypertension and hyperlipidaemia was higher in participants with T2D. Baseline characteristics, LA and LV parameters in T2D stratified by treatment group are presented in Supplemental Table S1. Overall, there were no significant differences in LA or LV parameters at baseline between the trial groups.

**Table 1:** Demographics, medical history, and medication of participants with T2D and controls

Parameter	T2D (n = 73)	Controls (n = 35)	*p*-value
Age, years	50.4 ± 6.3	48.6 ± 6.3	0.170
Sex, n (%) males	45 (61.6%)	19 (56.3%)	0.727
Height, cm	168.8 ± 9.4	169.3 ± 9.4	0.778
Weight, kg	102.6 ± 15.9	70.4 ± 10.9	< 0.001*
BMI, kg/m^2^	36.1 ± 5.3	24.5 ± 2.4	< 0.001*
SBP, mmHg	139.8 ± 15.1	121.2 ± 13.4	< 0.001*
DBP, mmHg	87.8 ± 7.6	76.5 ± 7.3	< 0.001*
HR, beats/min	74.4 ± 9.8	61.8 ± 9.9	< 0.001*
Medical history			
Diabetes duration, months	65.6 ± 39.1	N/A	N/A
Hypertension, n (%)	36 (49.3%)	0 (0)	< 0.001*
Hyperlipidaemia, n (%)	44 (60.3%)	0 (0)	< 0.001*
Fasting blood tests			
Glucose, mmol/L	8.4 ± 2.47	5.1 ± 0.48	< 0.001*
HbA1c, %	7.3 ± 1.03	5.4 ± 0.24	< 0.001*
Medications			
ACE inhibitor, n (%)	21 (28.8%)	0 (0)	< 0.001*
ARB, n (%)	10 (13.7%)	0 (0)	0.022*
Beta blocker, n (%)	4 (5.5%)	0 (0)	0.158
Calcium channel blocker, n (%)	16 (21.9%)	0 (0)	0.003*
Statin, n (%)	47 (64.4%)	0 (0)	< 0.001*
Metformin, n (%)	71 (97.3%)	N/A	N/A
Sulfonylurea, n (%)	11 (15.1%)	N/A	N/A
DPP-IV inhibitor, n (%)	14 (19.2%)	N/A	N/A
SGLT2 inhibitor, n (%)	9 (12.3%)	N/A	N/A
GLP-1 receptor agonist, n (%)	8 (11.0%)	N/A	N/A

Data represented as mean ± SD or number (%)

*SBP* systolic blood pressure, *DBP* diastolic blood pressure, *HR* heart rate, *ACEi* angiotensin converting enzyme inhibitor, *ARB* angiotensin-receptor blocker, *CCB* calcium channel blocker, *DPP-IV* dipeptidyl peptidase-IV, *SGLT2* sodium glucose cotransporter-2, *GLP-1* glucagon-like peptide-1

*Indicates a significant difference with p < 0.05

### Baseline imaging comparison between T2D and controls

Baseline LA and LV parameters for participants with T2D versus controls are shown in Table 2. In comparison to controls, T2D participants had significantly lower LV indexed volumes, higher LVEF and more concentric LV remodelling (higher mass:volume). Echocardiography suggested diastolic dysfunction (lower E/A) and higher LV filling pressures (E/e′) in T2D. LA indexed volumes and passive EF were lower in T2D than controls. However, active EF was higher in people with T2D, resulting in no difference in total LAEF between the two groups. Both reservoir and conduit LAS were lower in people with T2D (31.4 ± 7.4 *vs* 39.8 ± 10.8%, p < 0.001 and 15.9 ± 5.5 *vs* 24.1 ± 9.5%, p < 0.001, respectively). There was no difference in booster pump LAS between groups.

**Table 2:** Baseline LA and LV assessment parameters of participants with T2D versus controls

Parameter	T2D (n = 73)	Controls (n = 35)	*p*-value
Cardiac magnetic resonance imaging			
Volumetric assessment			
LAVi_Max_, ml/m^2^	33.7 ± 8.0	43.3 ± 10.8	< 0.001*
LAVi_Min_, ml/m^2^	14.9 ± 5.2	18.6 ± 5.7	0.002*
LA total EF, %	56.4 ± 7.6	57.3 ± 5.0	0.508
LA passive EF, %	27.4 ± 8.5	34.5 ± 7.8	< 0.001*
LA active EF, %	39.9 ± 7.7	34.6 ± 6.6	0.001*
LV EDVi, ml/m^2^	67.7 ± 10.2	83.2 ± 18.9	< 0.001*
LV ESVi, ml/m^2^	21.9 ± 6.4	29.5 ± 9.1	< 0.001*
LV EF, %	68.0 ± 6.8	65.0 ± 4.9	0.012*
LV mass, g	123.0 ± 24.6	107.0 ± 32.8	0.014*
LV mass index, g/m^2^	55.9 ± 8.7	58.1 ± 13.8	0.381
LV mass/volume, g/ml	0.83 ± 0.11	0.70 ± 0.10	< 0.001*
LA strain			
LAS_r, %	31.4 ± 7.4	39.8 ± 10.8	< 0.001*
LAS_cd, %	15.9 ± 5.5	24.1 ± 9.5	< 0.001*
LAS_bp,%	15.5 ± 4.9	15.6 ± 5.4	0.867
Echocardiography			
E-wave, m/s	0.67 ± 0.13	0.66 ± 0.13	0.820
A-wave, m/s	0.71 ± 0.15	0.56 ± 0.11	< 0.001*
E/A ratio	0.96 ± 0.19	1.21 ± 0.25	< 0.001*
Average E/e′ ratio	8.69 ± 2.5	6.40 ± 1.6	< 0.001*

Data represented as mean ± SD

*LAVi*_*max*_ left atrial maximum volume index, *LAVi*_*min*_ left atrial minimum volume index, *LAEF* left atrial emptying fraction, *LV EDVi* left ventricular end-diastolic volume index, *LV ESVi* left ventricular end-systolic volume index, *LVEF* left ventricular ejection fraction, *LAS_r* left atrial strain at reservoir phase, *LAS_cd* Left atrial strain at conduit phase, *LAS_bp* left atrial strain at booster pump phase

*Indicates a significant difference with p < 0.05

### Change in anthropometrics and LV parameters post-lifestyle intervention

Changes in anthropometric and LV parameters from baseline to week-12 are shown in Supplemental Table S2. The MRP group demonstrated a significant reduction in weight, BMI, fasting glucose, HbA1c and systolic blood pressure (SBP), whilst there were no significant changes noted in the exercise group. SBP was also reduced in the standard care group, driven by up-titration of guideline-based antihypertensive medications. There were no significant changes in the LV volumes, EF or mass in the standard care or exercise groups. The MRP group showed a significant increase in LV end-diastolic volume index (LVEDVi), with a corresponding decrease in LVEF, though remaining within normal range.

### Change in LA parameters post-lifestyle intervention

**Fig. 3:**
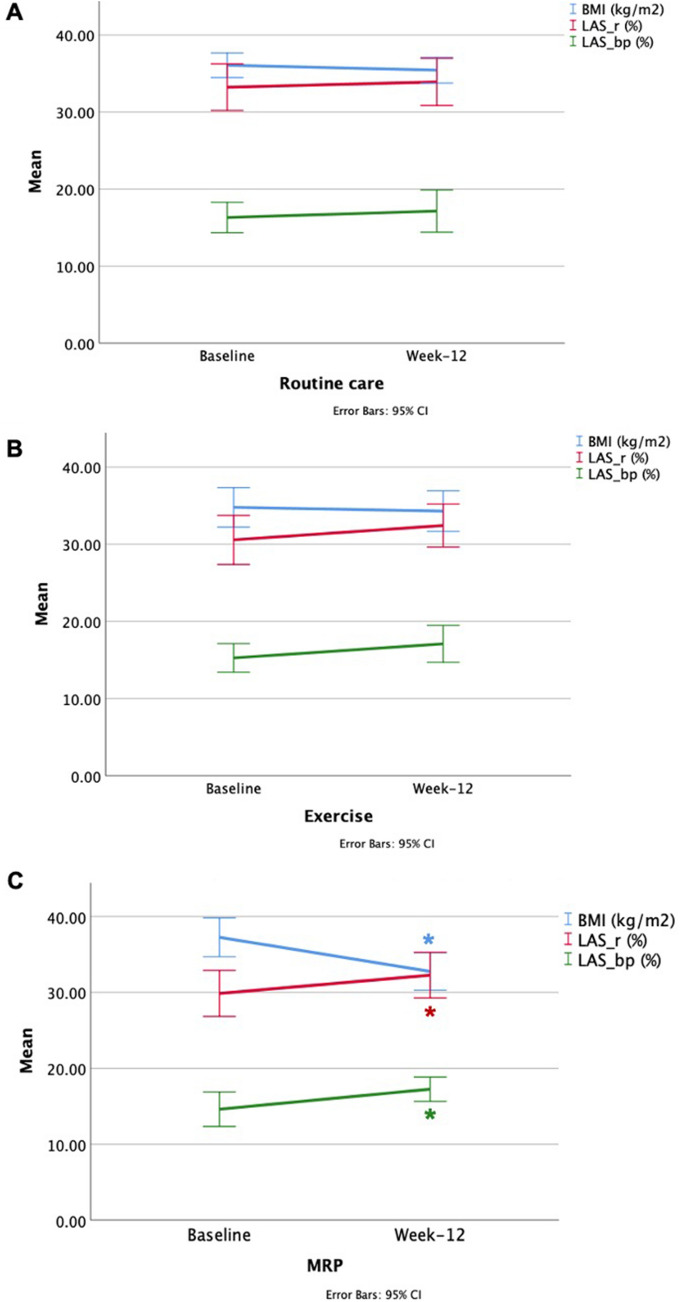
Changes in BMI, reservoir and booster-pump LAS in participants with T2D after 12 weeks of lifestyle intervention. Line graph representing change of body mass index (BMI) (blue), left atrial strain at reservoir (LAS_r) (Red), and left atrial strain at booster-pump (LAS_bp) (Green). Changes post lifestyle intervention (x-axis) at baseline and week-12 post routine care (**A**), exercise (**B**) and meal replacement plan (MRP) (**C**). Means are shown with error bars depicting standard error of the mean (y-axis) (*Indicates a significant difference with p < 0.05)

The LA volumetric and strain parameters by CMR at baseline and 12 weeks are shown in Table 3. On between-group analysis, corrected for baseline values, there were no statistically significant changes for any LA parameter, relative to the standard care group (p > 0.117). However, within-group analysis showed a significant increase in the maximal LAVi of borderline significance in the MRP group (36.2 ± 9.2 to 40.2 ± 13.4 mL/m^2^, p = 0.06). The MRP group also demonstrated a statistically significant increase in LAS at both reservoir and booster pump phases (29.9 ± 7.0 to 32.3 ± 7.0%, p = 0.036 and 14.6 ± 5.3 to 17.2 ± 3.7%, p = 0.034, respectively) (Fig. 3). There was no change in any strain parameter in the standard care group, and a trend towards an increase in the booster-pump LAS in the exercise group (15.3 ± 4.2 to 17.1 ± 5.4%, p = 0.09).

**Table 3:** The change in LA volumetric and strain parameters by CMR from baseline to week-12 in the three trial groups

LA parameter	Routine care (n = 28)				Exercise (n = 22)					MRP (n = 23)				
	Baseline	Week 12	*p*-value	Mean difference (95% CI)	Baseline	Week 12	*p*-value	Mean difference (95% CI)	Intervention effect (p-value)	Baseline	Week 12	*p*-value	Mean difference (95% CI)	Intervention effect (p-value)
LA volumetric assessment														
LAV_Max_, ml	70.5 ± 16.6	70.1 ± 20.6	0.84	− 0.41 (− 4.58, 3.74)	70.4 ± 17.1	71.1 ± 16.5	0.77	0.66 (− 3.99, 5.31)	0.725	81.3 ± 22.1	83.8 ± 27.7	0.51	2.51 (− 5.33, 10.3)	0.491
LAVi_Max_, ml/m^2^	32.4 ± 6.7	32.6 ± 9.3	0.87	0.20 (− 2.30, 2.69)	32.9 ± 8.0	33.4 ± 7.4	0.73	0.46 (− 2.26, 3.18)	0.840	36.2 ± 9.2	40.2 ± 13.4	0.06	3.98 (− 0.11, 8.08)	0.117
LAV_Min_, ml	29.4 ± 9.3	28.9 ± 9.8	0.61	− 0.59 (− 2.96, 1.77)	31.3 ± 10.9	31.1 ± 8.1	0.91	− 0.21 (− 3.95, 3.53)	0.573	37.8 ± 14.2	37.4 ± 15.4	0.88	− 0.37 (− 5.31, 4.57)	0.454
LAVi_Min_, ml/m^2^	13.5 ± 3.7	13.4 ± 4.3	0.86	− 0.10 (− 1.26, 1.05)	14.6 ± 5.2	14.6 ± 3.6	0.97	− 0.03 (− 1.92, 1.85)	0.555	16.8 ± 6.2	17.9 ± 7.6	0.35	1.13 (− 1.34, 3.61)	0.178
LA total EF, %	58.4 ± 6.2	58.9 ± 4.9	0.69	0.48 (− 2.01, 2.97)	55.8 ± 9.2	56.0 ± 5.9	0.91	0.20 (− 3.33, 3.74)	0.141	54.5 ± 7.2	56.5 ± 6.8	0.16	2.03 (− 0.85, 4.91)	0.603
LA passive EF, %	28.3 ± 7.0	30.0 ± 78	0.29	1.65 (− 1.52, 4.82)	28.2 ± 11.0	30.0 ± 8.6	0.47	1.80 (− 3.32, 6.92)	0.962	25.5 ± 7.4	28.1 ± 7.5	0.14	2.56 (− 0.92, 6.04)	0.743
LA active EF, %	42.0 ± 6.4	41.0 ± 6.8	0.54	− 1.04 (− 4.47, 2.38)	38.4 ± 9.0	36.5 ± 9.1	0.29	− 1.88 (− 5.49, 1.74)	0.166	38.9 ± 7.4	39.4 ± 8.5	0.81	0.52 (− 3.97, 5.01)	0.618
LA strain assessment														
LAS_r, %	33.2 ± 7.8	33.9 ± 7.9	0.72	0.68 (− 3.17,4.53)	30.6 ± 7.2	32.4 ± 6.3	0.28	1.86 (− 1.64, 5.36)	0.674	29.9 ± 7.0	32.3 ± 7.0	0.036*	2.41 (− 0.70, 5.52)	0.770
LAS_cd, %	16.9 ± 6.2	16.8 ± 5.3	0.90	− 0.15 (− 2.58, 2.28)	15.3 ± 5.2	15.3 ± 3.7	0.98	0.04 (− 2.49, 2.57)	0.453	15.3 ± 4.7	15.0 ± 5.3	0.86	− 0.23 (− 3.05, 2.58)	0.397
LAS_bp, %	16.3 ± 5.1	17.1 ± 7.1	0.95	0.83 (− 2.14, 3.80)	15.3 ± 4.2	17.1 ± 5.4	0.09	1.82 (− 0.30, 3.95)	0.808	14.6 ± 5.3	17.2 ± 3.7	0.034*	2.64 (0.21, 5.07)	0.731

Data represented as mean ± SD

Intervention effect = The change relative to routine care adjusted for baseline

*LAVi*_*max*_ left atrial maximum volume index, *LAVi*_*min*_ left atrial minimum volume index, *LAEF* left atrial emptying fraction, *LAS_r* Left atrial strain at reservoir phase, *LAS_cd* left atrial strain at conduit phase, *LAS_bp* Left atrial strain at booster pump phase, *MRP* meal replacement plan (~ 810 kcal/day)

*Indicates a significant difference with p < 0.05

### Correlation

Our previous publication showed significant correlations between volumetric and strain parameters corresponding to LA phasic function [26]. In this study, we investigated the association between the diastology parameters by echocardiography and LA function parameters by CMR. There were significant correlations of average-e′ with conduit LAS and passive LAEF, and A-wave with booster-pump LAS (Supplemental Fig. S1).

## Discussion

To our knowledge, this is the first study to investigate the impact of lifestyle interventions on LA strain parameters in adults with T2D and obesity. On within-group analysis, a low-energy MRP led to significant reductions in BMI, BP and hyperglycaemia, with a corresponding significant increase in reservoir and booster-pump LAS, despite no significant change in conventional LA volumetric parameters or echocardiographic measures of diastolic function. However, these changes were no longer significant when between-group interaction was taken into account.

### LA volumes and function in T2D

Previous LAV data in people with T2D are conflicting, with some studies showing larger LA volumes compared to controls [11, 27], whilst others show it to be smaller [28, 29]. These findings may reflect duration of disease as well as the effect of indexing volumes. In our study, participants with T2D had lower LAVi than controls, in line with previous studies comparing adults with and without T2D from UK Biobank [28] and in heart failure with preserved ejection fraction (HFpEF) patients with T2D [30, 31].

LA function is recognized as a predictor of HF hospitalization and adverse outcomes across a range of cardiovascular diseases [32–36]. Our results show people with T2D had lower passive LAEF corresponding to LA conduit function, which may reflect reduced LV compliance [10, 37]. This was in conjunction with impaired LV relaxation as mitral E/A was also lower. Consequently, active LAEF was higher in people with T2D to compensate for the reduction in passive LAEF, as shown previously [37]. This phenomenon has also been seen in early stages of hypertensive heart disease [38], however, absent in cases where the LV filling pressure is chronically elevated such as in HFpEF [39]. Accordingly, we observed that our asymptomatic participants with T2D were at early stages of LV diastolic dysfunction with grade-1 LV diastolic dysfunction.

People with T2D also showed impaired conduit LAS. In addition, LA filling was reduced as measured by a reduction in reservoir LAS. This finding may support previous studies where LAS detects subclinical reservoir dysfunction in people with T2D, even in those with normal LA volume, suggesting an early impairment in LA reservoir function [10, 40]. A previous study also demonstrated lower reservoir and conduit LAS in younger adults with obesity compared with normal-weight volunteers [41].

### Changes in LA parameters post-lifestyle intervention

Our results showed no significant change in LA volumes and EF measured by CMR in any of the trial groups on both between- and within-group analysis. However, LAS showed a within-group increase in the reservoir and booster-pump function in the MRP group only, in combination with a significant reduction in BMI and SBP. As these changes were not statistically significant on between-group analysis, they should be considered hypothesis generating, and could suggest an improvement in LA filling and contractility as a result of the low-calorie diet. The improvement in LA reservoir function following MRP could be explained by increased LA compliance. Studies have shown LV diastolic function improves after weight loss in people with obesity after 6-months of a lifestyle intervention [42, 43]. The current study did not demonstrate an improvement of LV diastolic function in the MRP group, which could be attributed to the short duration. The improvement in the booster pump LAS could be attributed to the Frank-Starling mechanism and the increase of preload as LA filling at reservoir was improved [44–46]. Another possible explanation for this might be the presence of a relationship between LA compliance and excess body weight, possibly linked to myocardial fat accumulation and systemic inflammation. Significant weight loss following the MRP likely contributed to these changes. A recent study compared participants with T2D, only participants with T2D and obesity had significantly lower LAS at reservoir and booster-pump [47].

Together, the present findings confirm that early changes in LA reservoir function assessed by strain may precede changes in conventional volumetric measures in people with T2D and obesity. Weight loss via a low energy MRP could improve LA reservoir function. Although, aerobic exercise showed no significant change in LA function, we previously reported an improvement in LV-PEDSR. Therefore, further studies are needed to investigate the combination of exercise and diet to achieve significant weight loss that may provide optimal reversal of diastolic function, with potential prevention HF in people with T2D and obesity.

### Left atrial strain as an imaging biomarker

The findings of this study suggest that reservoir LAS could be a useful non-invasive marker to detect early LA dysfunction and more interestingly, improvement post-intervention in people with T2D and obesity. Accordingly, if backed by future studies, LAS may have the potential to be used as outcome measure in clinical trials. Moreover, it could be used in combination with LV strain assessment to detect subclinical impairments in cardiac function and provide an opportunity for early intervention to prevent disease progression.

### Study limitations

The DIASTOLIC study limitations have been previously published and included the small sample size, unblinded intervention, short duration of follow-up and the high rate (19%) of non-compliance in the exercise group [23]. Although the DIASTOLIC study achieved the trial statistical power, it could be under powered for this post-hoc analysis, which limits the detection of between-groups differences. However, these analyses should be considered hypothesis-generating, and further studies are needed to confirm these novel findings.

## Conclusion

In working-age adults with T2D and obesity, a 12-week lifestyle intervention of low-energy MRP, but not exercise training, led to significant improvements in LA reservoir and booster-pump function assessed by CMR LAS, in conjunction with a significant reduction in BMI and SBP. However, these were not significant on between-group analysis, and should be considered hypothesis-generating.

### Supplementary Information

Below is the link to the electronic supplementary material.

Supplementary file1 (DOCX 27 KB)

Supplementary file2 (JPG 160 KB)

## Data Availability

ClinicalTrials.gov Identifier: NCT02590822.

## Abbreviations

LA: Left atrium
LAEF: Left atrial emptying fraction
LAS _bp: Left atrial strain at booster pump phase
LAS_cd: Left atrial strain at conduit phase
LAS_r: Left atrial strain at reservoir phase
LAV (max/min/pre-A): Left atrial volume (maximal/minimal/pre-atrial contraction)
T2D: Type 2 diabetes
