# Supplementary material for: The impact of lifestyle intervention on left atrial function in type 2 diabetes: results from the DIASTOLIC study
Source: Int J Cardiovasc Imaging. 2022 Mar 2;38(9):2013–23. doi: 10.1007/s10554-022-02578-z (PMC10247829; doi:10.1007/s10554-022-02578-z)
Supplement: Supplementary file 1 — Supplementary file1 (DOCX 27 KB) [file 10554_2022_2578_MOESM1_ESM.docx]

**Online only Supplementary**

**Tables:**

**Supplemental Table_S1:** Baseline characteristics, left atrium and left ventricle parameters in people with T2D stratified by treatment group.

| Parameter | Routine care (n=28) | Exercise (n=22) | MRP (n=23) |
| --- | --- | --- | --- |
| **Age, years** | 50.1±6.1 | 50.1±7.3 | 51.0±5.8 |
| **Sex, M (%)** | 18 (64.0%) | 13 (59.10%) | 14 (60.9%) |
| **Height, cm** | 168.0±10.3 | 169.0±8.5 | 169.5±9.5 |
| **Weight, kg** | 101.8±15.0 | 99.2±16.3 | 106.7±16.3 |
| **BMI, kg/m^2^** | 36.1±4.2 | 34.8±5.8 | 37.3±5.9 |
| **SBP, mmHg** | 138.8±12.4 | 135.5±16.9 | 145.1±15.3 |
| **DBP, mmHg** | 86.0±7.0 | 87.2±8.2 | 90.6±7.2 |
| **HR, beats/min** | 75.9±7.5 | 75.0±12.7 | 72.0±9.0 |
| **FASTING BLOOD TESTS** | | | |
| **Glucose, mmol/L** | 8.3±2.40 | 8.6±2.49 | 8.3±2.62 |
| **HbA1c, %** | 7.3±0.95 | 7.4±1.11 | 7.2±1.08 |
| **Echocardiography** | | | |
| **E-wave, m/s** | 0.69±0.15 | 0.65±0.12 | 0.65±0.12 |
| **A-wave, m/s** | 0.69±0.16 | 0.71±0.16 | 0.72±0.12 |
| **E/A ratio** | 1.01±0.19 | 0.94±0.19 | 0.92±0.20 |
| **Average E/e’ ratio** | 8.5±2.3 | 8.6±2.6 | 9.1±2.7 |
| **Cardiac Magnetic Resonance Imaging** | | | |
| ***Volumetric assessment*** | | | |
| **LAVi_Max_, ml/m^2^** | 32.4±6.7 | 32.9±8.0 | 36.2±9.2 |
| **LAVi_Min_, ml/m^2^** | 13.5±3.7 | 14.6±5.2 | 16.8±6.2 |
| **LA total EF, %** | 58.4±6.2 | 55.8±9.2 | 54.5±7.2 |
| **LA passive EF, %** | 28.3±7.0 | 28.2±11.0 | 25.5±7.4 |
| **LA active EF, %** | 42.0±6.4 | 38.4±9.0 | 38.9±7.4 |
| **LV EDVi, ml/m^2^** | 65.3±10.5 | 67.8±8.2 | 70.6±11.0 |
| **LV ESVi, ml/m^2^** | 21.3±5.1 | 22.7±7.0 | 21.7±7.5 |
| **LV EF, %** | 67.4±5.2 | 66.8±7.9 | 69.8±7.4 |
| **LV mass, g** | 116.1±23.2 | 123.1±21.9 | 131.2±26.9 |
| **LV mass index, g/m^2^** | 53.1±7.7 | 57.0±7.5 | 58.3±10.1 |
| **LV mass/volume, g/ml** | 0.82±0.11 | 0.85±0.12 | 0.83±0.13 |
| ***LA Strain*** | | | |
| **LAS_r, %** | 33.2±7.8 | 30.6±7.2 | 29.9±7.0 |
| **LAS_cd, %** | 16.9±6.2 | 15.3±5.2 | 15.3±4.7 |
| **LAS_bp,%** | 16.3±5.1 | 15.3±4.2 | 14.6±5.3 |

Data represented as mean ± SD or number (%).

Abbreviations: SBP= systolic blood pressure, DBP= diastolic blood pressure, HR= heart rate, LAVi_max_= Left atrial maximum volume index, LAVi_min_= Left atrial minimum volume index, LAEF= left atrial emptying fraction, LV EDVi= left ventricular end-diastolic volume index, LV ESVi= left ventricular end-systolic volume index LVEF= left ventricular ejection fraction, LAS_r = Left atrial strain at reservoir phase, LAS_cd = Left atrial strain at conduit phase, LAS_bp = Left atrial strain at booster pump phase.

**Supplemental Table_S2:** The change in anthropometric and LV parameters from baseline to week-12 in the three trial groups.

|  | Routine care (n=28) | | | | Exercise (n=22) | | | | MRP (n=23) | | | | | |
| --- | --- | --- | --- | --- | --- | --- | --- | --- | --- | --- | --- | --- | --- | --- |
|  | **Baseline** | **Week 12** | **p-value** | **Mean difference (95% CI)** | **Baseline** | **Week 12** | **p-value** | **Mean difference (95% CI)** | **Baseline** | **Week 12** | | **p-value** | | **Mean difference (95% CI)** |
| Weight, kg | 101.8±15.0 | 100.1±14.9 | 0.652 | -1.78  (-9.78,6.23) | 99.2±16.3 | 97.8±16.6 | 0.737 | -1.38  (-9.83,7.07) | 106.7±16.3 | 93.5±15.1 | | **<0.001*** | | -13.2  (-19.3,-7.02) |
| BMI, kg/m^2^ | 36.1±4.2 | 35.4±4.3 | 0.581 | -0.63  (-2.93,1.68) | 34.8±5.8 | 34.3±5.9 | 0.740 | -0.49  (-3.53,2.54) | 37.3±5.9 | 32.8±5.7 | | **<0.001*** | | -4.50  (-5.97,-3.03) |
| SBP, mmHg | 138.8±12.4 | 131.6±14.6 | **0.031** | -7.17  (-13.6,-0.71) | 135.5±16.9 | 133.0±14.3 | 0.550 | -2.45  (-10.9,5.95) | 145.1±15.3 | 133.3±18.2 | | **0.015*** | | -11.7  (-20.9,-2.54) |
| DBP, mmHg | 86.0±7.0 | 84.5±9.9 | 0.526 | -1.50  (-6.29.3.29) | 87.2±8.2 | 86.7±8.5 | 0.778 | -0.55  (-4.51,3.42) | 90.6±7.2 | 86.9±9.0 | | 0.181 | | -3.74  (-9.35,1.87) |
| HR, beats/min | 75.9±7.5 | 74.1±9.5 | 0.324 | -1.86  (-5.65,1.93) | 75.0±12.7 | 73.4±9.6 | 0.545 | -1.55  (-6.77,3.68) | 72.0±9.0 | 68.1±9.8 | | 0.205 | | -3.87  (-10.0,2.28) |
| FASTING BLOOD TESTS | | | | | | | | | | | | | | |
| Glucose, mmol/L | 8.3±2.40 | 8.1±2.35 | 0.724 | 0.15  (-0.70,1.00) | 8.6±2.49 | 7.7±1.89 | 0.075 | 1.24  (-0.14,2.62) | 8.3±2.62 | 6.4±1.27 | | **<0.001*** | | 2.21  (1.00,3.42) |
| HbA1c, % | 7.3±0.95 | 7.2±1.09 | 0.664 | 0.06  (-0.21,0.32) | 7.4±1.11 | 7.3±1.13 | 0.360 | 0.10  (-0.13,0.34) | 7.2±1.08 | 6.2±0.68 | | **<0.001*** | | 0.97  (0.55,1.39) |
| Cardiac Magnetic Resonance Imaging | | | | | | | | | | | | | | |
| LV EDVi, ml/m^2^ | 65.3±10.5 | 65.4±13.4 | 0.962 | 0.13  (-5.51,5.78) | 67.8±8.2 | 69.3±9.2 | 0.479 | 1.50  (-2.82,5.81) | 70.6±11.0 | 75.6±14.2 | | 0.227 | | 4.97  (-3.33,13.3) |
| LV ESVi, ml/m^2^ | 21.3±5.1 | 22.3±6.9 | 0.582 | 0.92  (-2.48,4.33) | 22.7±7.0 | 23.7±5.8 | 0.511 | 0.94  (-1.98,3.85) | 21.7±7.5 | 26.5±7.8 | | **0.016*** | | 4.79  (1.00,8.59) |
| LV EF, % | 67.4±5.2 | 66.4±5.2 | 0.530 | -1.02  (-4.26,2.23) | 66.8±7.9 | 66.0±6.2 | 0.613 | -0.79  (-3.96,2.38) | 69.8±7.4 | 65.2±6.1 | | **0.003*** | | -4.54  (-7.41,-1.66) |
| LV mass, g | 116.1±23.2 | 116.6±25.1 | 0.920 | 0.54  (-10.3,11.3) | 123.1±21.9 | 122.0±20.9 | 0.754 | -1.15  (-8.66,6.36) | 131.2±26.9 | 125.6±27.0 | | 0.495 | | -5.56  (-22.2,11.1) |
| LV mass index, g/m^2^ | 53.1±7.7 | 53.7±8.3 | 0.699 | 0.66  (-2.79,4.11) | 57.0±7.5 | 56.9±7.9 | 0.942 | -0.10  (-3.18,2.97) | 58.3±10.1 | 59.8±10.7 | | 0.613 | | 1.56  (-4.76,7.87) |
| LV mass/volume, g/ml | 0.82±0.11 | 0.84±0.14 | 0.640 | 0.02  (-0.05,0.09) | 0.85±0.12 | 0.83±0.12 | 0.487 | -0.02  (-0.08,0.04) | 0.83±0.13 | 0.80±0.11 | | 0.300 | | -0.03  (-0.09,0.03) |
| Echocardiography | | | | | | | | | | | | | | |
| E/A ratio | 1.01±0.19 | 1.02±0.25 | 0.806 | 0.01  (-0.10,0.12) | 0.94±0.19 | 1.0±0.21 | 0.253 | 0.06  (-0.05,0.16) | 0.92±0.20 | | 1.0±0.22 | 0.358 | 0.10  (-0.05,0.24) | |
| Average E/e’ ratio | 8.5±2.3 | 8.3±1.9 | 0.711 | -0.13  (-0.83,0.58) | 8.6±2.6 | 8.6±2.5 | 0.870 | -0.11  (-1.47,1.25) | 9.1±2.7 | | 8.6±1.7 | 0.424 | -0.41  (-1.46,0.64) | |

Data represented as mean ± SD

* indicates a significant difference with p<0.05

Abbreviations: SBP= systolic blood pressure, DBP= diastolic blood pressure, HR= heart rate, LV EDVi= left ventricular end-diastolic volume index, LV ESVi= left ventricular end-systolic volume index LVEF= left ventricular ejection fraction, MRP = meal replacement plan (~801kcal/d).

**Figures:**

**Supplemental Figure_S1**: **Associations of left atrial strain and emptying fractions parameters by CMR with LV diastolic parameters by echocardiogram in T2D participants.**

Caption: Transmitral E-wave and Average-e’ by tissue Doppler Echocardiography corresponded to LA strain at conduit (LAS_cd) (A&B ,respectively). Transmitral E-wave and Average-e’ corresponded to passive left atrial emptying fraction (EF)(C&D ,respectively). Transmitral A-wave corresponded to active left atrial EF and LA strain at booster-pump (LAS_bp) (E&F ,respectively).
