# Supplementary figures and images for: The impact of lifestyle intervention on left atrial function in type 2 diabetes: results from the DIASTOLIC study
Source: Int J Cardiovasc Imaging. 2022 Mar 2;38(9):2013–23. doi: 10.1007/s10554-022-02578-z (PMC10247829; doi:10.1007/s10554-022-02578-z)

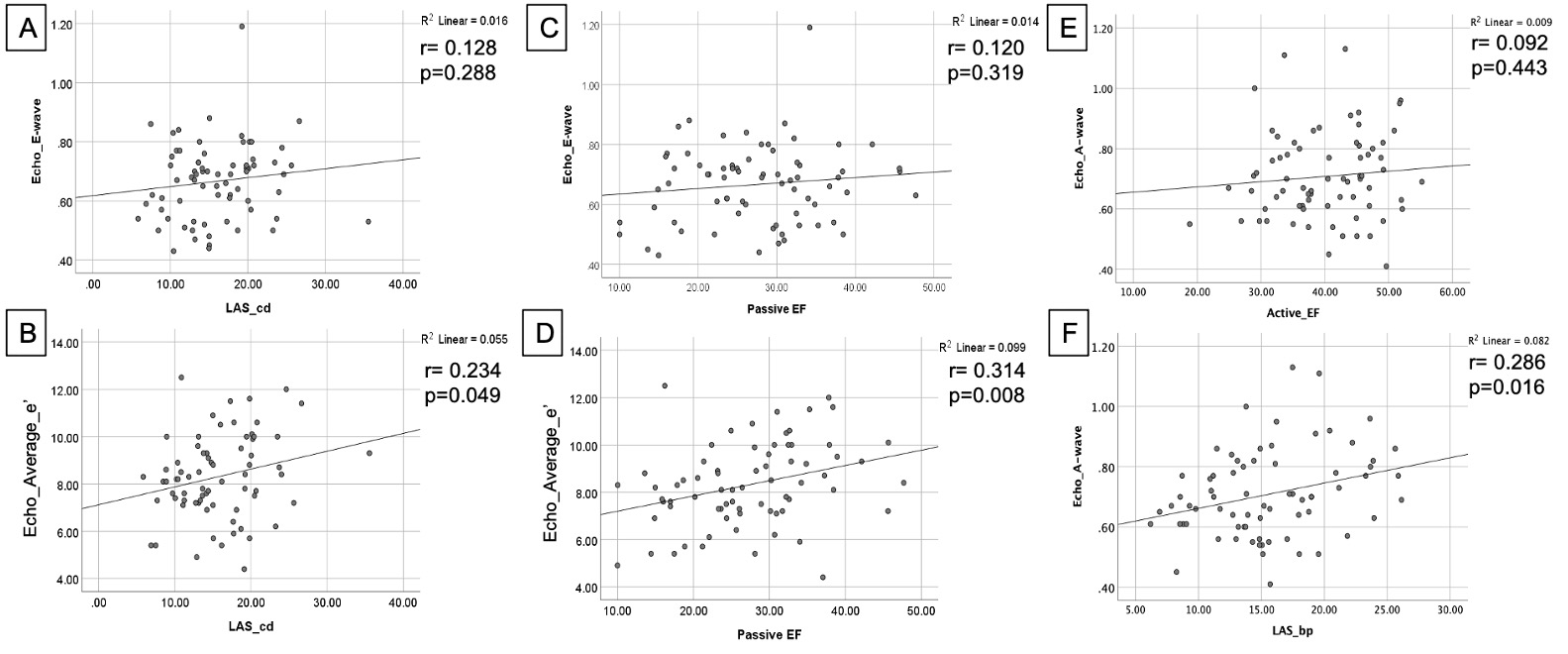

Supplement: Supplementary file 2 — Supplementary file2 (JPG 160 KB) [file 10554_2022_2578_MOESM2_ESM.jpg]
